# Artificial intelligence for detecting autism spectrum disorder: a systematic review and meta-analysis

**DOI:** 10.3389/fpubh.2026.1923851

**Published:** 2026-10-12

**Authors:** Qian Wang, Rong Luo, Qianhui Wen

**Affiliations:** 1 Department of Pediatrics, West China Second University Hospital, Sichuan University, Chengdu, Sichuan, China; 2 Key Laboratory of Birth Defects and Related Diseases of Women and Children (Sichuan University), Ministry of Education, Chengdu, Sichuan, China

**Keywords:** artificial intelligence, autism spectrum disorder, diagnosis, machine learning, meta-analysis

## Abstract

**Background:**

Autism spectrum disorder (ASD) is a heterogeneous neurodevelopmental condition, and early identification is essential for timely intervention. Conventional diagnosis relies on clinical interviews, behavioral observations, caregiver reports, and standardized tools, which are time-consuming and resource-dependent. Artificial intelligence (AI) offers opportunities for screening and adjunctive diagnosis, but studies vary in data sources, model types, and validation methods, requiring systematic evaluation.

**Methods:**

We searched PubMed, Embase, the Cochrane Library, Web of Science, and IEEE Xplore for studies of AI-based ASD detection. Eligible studies were required to provide ASD-versus-non-ASD diagnostic-accuracy data that could be extracted or reliably reconstructed at the independent participant or case level. Risk of bias and applicability were assessed using a modified QUADAS-AI tool. Pooled sensitivity and specificity were estimated with a bivariate Reitsma random-effects model. HSROC AUCs were calculated with bias-corrected and accelerated (BCa) 95% confidence intervals based on 5,000 study-level bootstrap replicates and were omitted when SROC fits were unstable. Sensitivity analyses addressed dataset overlap, model selection, and reference-standard quality.

**Results:**

Of 17,387 records, 26 studies were included. Pooled sensitivity was 0.908 (95% CI: 0.861–0.940), pooled specificity was 0.868 (95% CI: 0.830–0.898), and the HSROC AUC was 0.936 (BCa 95% CI: 0.860–0.973). A dataset-independent analysis of 21 studies yielded lower estimates (sensitivity 0.870, specificity 0.841, AUC 0.900). Leave-one-out results were generally stable. Funnel-plot asymmetry was not significant in the primary analysis and varied in direction and statistical significance across sensitivity analyses.

**Conclusions:**

AI-based models showed relatively high pooled diagnostic performance for ASD detection, but substantial heterogeneity, methodological and applicability concerns, limited external validation, and potential non-independence of underlying datasets limit the certainty and generalizability of the evidence. Funnel-plot analyses did not provide consistent evidence of conventional small-study effects or selective reporting. AI should therefore be considered an adjunct rather than a standalone diagnostic tool. Prospective multicenter validation in representative populations, participant-level evaluation, clearly defined reference diagnoses, prespecified models and operating thresholds, and independent evaluation datasets are needed before broader clinical implementation.

## 1 Introduction

Autism spectrum disorder (ASD) is a neurodevelopmental condition characterized by persistent deficits in social communication and social interaction, together with restricted interests and repetitive or stereotyped patterns of behavior (1). ASD is clinically heterogeneous, with substantial inter-individual variation in symptom severity, language and cognitive abilities, sensory and motor features, co-occurring conditions, and functional impairment. This heterogeneity complicates early identification and accurate diagnosis (2).

The reported prevalence of ASD has increased over recent decades. Globally, epidemiological evidence suggests that approximately 1 in 100 children is affected by ASD (3). In Asia, particularly Southeast Asia, prevalence estimates are lower, at approximately 6 per 1,000 individuals (4). In contrast, 2022 surveillance data from the US Autism and Developmental Disabilities Monitoring (ADDM) Network reported a prevalence of 32.2 per 1,000 among 8-year-old children across 16 monitoring sites, equivalent to approximately 1 in 31 children identified with ASD (5). Nevertheless, prevalence estimates differ across studies, owing to variations in study period, age range, diagnostic criteria, screening approaches, and surveillance systems. These trends, together with the growing demand for ASD-related services, highlight the need for efficient, objective, and scalable strategies to support ASD screening and diagnosis.

Early identification of ASD is essential for enabling timely intervention and improving developmental outcomes. Detecting ASD-related risks in early childhood and initiating prompt intervention may alleviate core symptoms and enhance social communication, adaptive functioning, and long-term development (6–8). However, ASD cannot be confirmed by a single laboratory test, biomarker, or neuroimaging examination. Its diagnosis relies primarily on multidisciplinary clinical assessment, including clinical interviews, behavioral observations, caregiver reports, and standardized diagnostic instruments. Although these approaches remain the diagnostic standard, they are time-intensive, resource-demanding, and dependent on highly trained professionals, which limits their accessibility in primary care, educational, and resource-constrained settings (9, 10). Early recognition and differential diagnosis are further complicated by developmental changes in ASD symptoms and frequent co-occurrence with conditions such as attention-deficit/hyperactivity disorder, language delay, intellectual disability, and anxiety disorders (9). These challenges underscore the need for efficient, objective, and scalable tools to support ASD screening and diagnostic prediction.

Artificial intelligence (AI) has increasingly been applied to ASD screening, diagnostic support, and risk prediction. Machine learning and deep learning models are capable of extracting latent representations from high-dimensional and multimodal data, enabling the identification of ASD-related patterns. Prior studies have reported promising predictive performance using approaches such as support vector machines (SVM), k-nearest neighbors (KNN), and deep learning (DL) for ASD classification and diagnosis (11–15). For instance, Kong et al. combined brain connectivity features with a deep neural network classifier for ASD classification (16), while Guo et al. (17) proposed a deep neural network-based feature selection method for childhood ASD diagnosis that outperformed conventional methods such as elastic net. In addition, EEG preprocessing, including artifact removal, is an important component of EEG-based ASD identification because preprocessing quality may affect subsequent feature extraction and classification (18). Collectively, these findings indicate that AI models can capture ASD-relevant patterns across diverse data sources and provide auxiliary support for ASD screening and diagnosis. Nevertheless, many existing models are constrained by task-specific designs, single-modality data, and relatively small sample sizes, and their generalizability and real-world applicability remain insufficiently validated.

AI-based studies on ASD screening and diagnostic prediction have incorporated a wide range of data modalities, including eye tracking, EEG, functional near-infrared spectroscopy (fNIRS), video, audio, motor behaviors, electronic health records, clinical text, and other biological or developmental measures (19–24). These modalities provide complementary information on the complex and heterogeneous ASD phenotype. In this context, multimodal AI approaches may be particularly valuable, as they integrate behavioral, neurophysiological, clinical, and developmental data to better characterize diverse ASD presentations. Beyond improving objectivity and scalability, AI-based prediction may facilitate the detection of subtle patterns that are difficult to capture through conventional assessments. Nevertheless, clinical utility depends not only on predictive accuracy but also on implementability, cost, accessibility, and feasibility in real-world settings. Although MRI and genetic testing can provide important neurobiological insights, their reliance on specialized equipment, standardized protocols, and trained personnel limits their applicability in primary screening, educational settings, and large-scale implementation.

Despite rapid advances in AI-based ASD screening and diagnostic prediction, the current evidence remains highly heterogeneous with respect to data modalities, model architectures, sample characteristics, validation strategies, and outcome reporting. In addition, some reported estimates of high diagnostic accuracy may be overoptimistic because of small sample sizes, exclusive reliance on internal validation, lack of clinical control groups, potential data leakage, and inconsistencies in the unit of analysis. A systematic review and meta-analysis is therefore warranted to synthesize existing evidence and quantitatively evaluate the diagnostic performance of relatively low-burden AI-assisted tools for ASD screening and diagnostic support. Accordingly, this study aims to systematically review and meta-analyze the diagnostic performance of such tools, while further assessing their applicability and scalability across data sources, model types, validation strategies, and potential clinical scenarios. These findings may inform the development and translation of AI-assisted approaches for early ASD screening, risk stratification, and clinical decision-making.

Accordingly, this review aims to provide a clinically oriented synthesis of AI-assisted ASD detection, with a focus on relatively low-burden and scalable approaches across behavioral, clinical, neurophysiological, eye-tracking/video-audio, retinal or facial imaging, and multimodal data. Beyond summarizing diagnostic performance, we critically evaluate the consistency, robustness, and generalizability of the available evidence across key study characteristics. By considering diagnostic performance alongside evidence quality and clinical applicability, this review seeks to clarify the current potential, limitations, and translational relevance of AI-assisted approaches for ASD screening and diagnostic support.

## 2 Methods

This systematic review and meta-analysis was designed and reported in accordance with the Preferred Reporting Items for Systematic Reviews and Meta-Analyses of Diagnostic Test Accuracy Studies (PRISMA-DTA) guidelines. The completed PRISMA-DTA checklist is provided in Supplementary Table S1. The review was registered with PROSPERO (CRD420251270564).

### 2.1 Inclusion criteria

Studies were included if they met the following criteria: (1) original research involving human participants; (2) development or validation of predictive models based on artificial intelligence, machine learning, or deep learning for the diagnosis, detection, or screening of ASD; (3) application of the model to ASD prediction or classification in comparison with a non-ASD group; (4) use of clinical, behavioral, EEG/fNIRS, eye-tracking, video, audio, imaging data, including retinal or facial imaging, or multimodal data; (5) inclusion of both ASD and non-ASD participants, with sufficient diagnostic accuracy data to extract or reconstruct a 2 × 2 contingency table; and (6) application of cross-validation, hold-out validation, internal validation, or external validation with reported diagnostic performance.

### 2.2 Exclusion criteria

Studies were excluded if they met any of the following criteria: (1) non-human studies, non-original research, or non-peer-reviewed publications; (2) non-English publications; (3) studies that did not develop or validate predictive models based on artificial intelligence, machine learning, or deep learning for ASD diagnosis, detection, or screening; (4) prediction or classification tasks that did not permit evaluation of ASD versus a non-ASD group for diagnostic-accuracy assessment, including population-surveillance case-ascertainment tasks that did not correspond to the clinically oriented ASD screening, detection, or diagnostic-prediction task of this review; (5) absence of non-ASD controls or insufficient diagnostic accuracy data to extract or reconstruct a 2 × 2 contingency table at the independent participant or case level; (6) studies reporting only training-set performance without cross-validation, hold-out validation, internal validation, or external validation; and (7) studies primarily based on structural or functional brain magnetic resonance imaging (MRI/fMRI), as the review was operationally focused on relatively low-burden and scalable approaches for ASD screening and diagnostic support.

The exclusion of studies primarily based on structural or functional brain MRI/fMRI was an operational restriction applied during review conduct and was not explicitly prespecified in the PROSPERO registration, which broadly listed neuroimaging among potentially eligible modalities.

### 2.3 Data sources and search strategy

We systematically searched PubMed, Embase, Web of Science, the Cochrane Library, and IEEE Xplore from inception to September 30, 2025, to identify original studies that applied artificial intelligence, machine learning, or deep learning methods for ASD diagnosis, detection, or screening, prediction, or classification. The search strategy combined controlled vocabulary terms, including MeSH and/or Emtree terms, with free-text keywords. No restrictions were applied on publication year or geographic region. Detailed search strategies for PubMed, Embase, Web of Science, the Cochrane Library, and IEEE Xplore are provided in Supplementary Table S2.

### 2.4 Study selection

Study selection was carried out in two stages based on the operational inclusion and exclusion criteria defined for this review. All records retrieved from the searches were first imported into EndNote. Duplicate records identified during reference management were removed before title and abstract screening. Two reviewers (QW and QHW) then independently screened the titles and abstracts of the remaining records to identify potentially eligible studies. The same reviewers independently assessed the full texts of potentially eligible articles to determine final eligibility. Any disagreements were resolved through discussion, and, when necessary, a third reviewer (RL) was consulted. For the final quantitative synthesis, all potentially eligible studies were additionally verified with particular attention to the unit of analysis and the availability of diagnostic-accuracy data at the participant or case level. Studies were excluded if the reported 2 × 2 data were based on repeated lower-level observations, such as images, signal segments, or sessions, rather than independent participants or cases. Studies were also excluded when the reported validation results did not allow a reliable participant- or case-level 2 × 2 contingency table to be extracted or reconstructed. Eligibility was further reassessed to ensure that the prediction task was consistent with the clinically oriented ASD screening, detection, or diagnostic-prediction scope of the review.

### 2.5 Data extraction, threshold handling, and model categorization

Data extraction was independently performed by two investigators (QW and QHW) using a predesigned standardized form. The following information was collected: first author, publication year, country where the study was conducted, study design, participant characteristics, participant-level cohort/sample size, unit of analysis, type of AI model, input variables, validation method, dataset source and potential dataset overlap, and model performance metrics, including sensitivity, specificity, accuracy, and the area under the receiver operating characteristic curve (AUC). Disagreements were resolved through discussion, and a third investigator (RL) was consulted when consensus could not be reached.

To facilitate structured comparisons across the included studies, the extracted characteristics were categorized by age group, data modality, algorithm family, and validation strategy. Age groups were classified as pediatric, adult/mixed-age, or unclear age. Data modality was defined according to the primary source of information used for ASD detection and categorized as clinical/behavioral, EEG/fNIRS, eye-tracking/video-audio, multimodal, or imaging. Among the 26 included studies, the EEG/fNIRS category was represented exclusively by EEG studies and is therefore reported as EEG in the subgroup analyses. Algorithms were classified according to their principal model family as support vector machine (SVM), ensemble methods other than random forest, decision tree, convolutional neural network (CNN), k-nearest neighbors (KNN), logistic regression, random forest, RNN/LSTM, or other/unclear. Validation strategy was classified according to the validation approach associated with the diagnostic-accuracy estimate retained for analysis as cross-validation, hold-out validation, external validation, or other/unclear. External validation referred to evaluation of the retained model in a dataset or population independent of the data used for model development and tuning. These categories informed the quantitative subgroup analyses and comparative narrative synthesis.

For classification models, we did not apply a uniform classification threshold across studies. Instead, we used the performance estimates reported in the original studies at their main operating thresholds. When multiple candidate thresholds were reported, we used the operating point designated by the original authors as primary, final, or recommended; otherwise, the operating point corresponding to the main validation or test result was used. If the threshold was not explicitly stated but sufficient data were provided to reconstruct a 2 × 2 contingency table, such as sensitivity, specificity, and the numbers of ASD and non-ASD participants, the reported estimates were regarded as reflecting the main operating point of the original study. Studies that reported only AUC, accuracy, or other overall performance measures, without enough information to extract or reconstruct a participant- or case-level 2 × 2 table, were excluded. For each included study, a 2 × 2 contingency table containing true positives, false negatives, false positives, and true negatives was extracted directly or reconstructed from the available data. When performance was reported only as an average across repeated cross-validation or repeated hold-out analyses, these summary estimates were not converted back into participant-level integer counts unless a reliable participant- or case-level 2 × 2 table could be established from the reported results. Only 2 × 2 tables representing participant- or case-level diagnostic classifications were used. When multiple images, signal segments, trials, or other repeated observations were available for the same participant, lower-level observations were not treated as independent diagnostic units; the diagnostic result had to be reported or reconstructable at the participant level. For studies reporting multiclass outputs, data were used only when the reported results permitted an unambiguous reconstruction of the binary ASD-versus-non-ASD classification at the participant level.

When a study reported more than one eligible AI model, we preferentially used the results from the model prespecified or explicitly designated by the original authors as the primary or final model. If no primary or final model was specified, we selected the best-performing eligible model or configuration from the validation or test phase based on the principal performance measure reported in the original study. For the model-selection sensitivity analysis, studies were classified into three mutually exclusive groups: (1) prespecified/author-designated primary or final model, in which the study-level estimate was derived from a model prespecified or explicitly identified by the original authors as primary or final; (2) reviewer-selected best-performing model, in which the review team selected the best-performing result from multiple eligible models or configurations because no primary or final model had been specified; and (3) single eligible model, in which only one model met the eligibility criteria and no model-selection decision was required.

This study-level selection framework was used to avoid within-study double-counting of participants across multiple model estimates or operating thresholds and to improve reproducibility when studies reported multiple models or thresholds. Potential non-independence across different reports was assessed separately using the reported dataset source or underlying cohort information. Reports drawing on the same named repository or on clearly or probably overlapping participant cohorts were flagged as potentially non-independent and documented in Table 1. Cross-study dataset overlap was addressed separately in the dataset-independent sensitivity analysis described in Section 2.7. The potential influence of review-level model selection on the pooled estimates was also examined in sensitivity analysis.

**Table 1:** Characteristics of the 26 included studies and participant-level analytic data.

No.	References	Country/region	Participant–level 2 × 2 analytic sample (ASD/total)	Participant–level cohort/sample size	Unit of analysis	Age group	Task	Data modality	Algorithm family	Validation strategy	Model–selection strategy	Reference standard	Dataset source	Overlap flag
1	Wall et al. (29)	USA	612/627	627 participants in primary classification (612 ASD, 15 non–spectrum); additional ASD–only validation cohorts reported	Participant/ADOS Module 1 record	Adult/Mixed	Diagnosis/detection	Clinical/Behavioral	Decision Tree	Cross–validation	Author–designated primary/final	Clearly defined	AGRE (development/primary); Boston Autism Consortium and SSC (additional validation)	Potential overlap: ADOS repositories
2	Duda et al. (26)	USA	789/807	807 participants in selected analysis (789 ASD, 18 non–ASD); 2,616 ADOS Module 1 records reported overall	Participant/ADOS Module 1 record	Pediatric	Diagnosis/detection	Clinical/Behavioral	Decision Tree	External validation	Single eligible model	Unclear/insufficient	AC/SSC/SVIP/AGRE/NDAR	Potential overlap: ADOS repositories
3	Kosmicki et al. (30)	USA	1,924/2,138	4,540 individuals across ADOS Modules 2/3; evaluation subset *n*=2,138	Participant/ADOS record	Pediatric	Diagnosis/detection	Clinical/Behavioral	Support Vector Machine	External validation	Reviewer–selected best–performing	Clearly defined	AC/SSC/AGRE/NDAR/SVIP	Potential overlap: ADOS repositories
4	Tariq et al. (31)	USA	33/66	66 children in prospective validation (33 ASD, 33 non–ASD); development cohort excluded from meta–analysis	Participant/validation video (1 per child)	Pediatric	Diagnosis/detection	Eye–tracking/video–audio	Logistic Regression	External validation	Author–designated primary/final	Unclear/insufficient	Crowdsourced/direct–upload/YouTube home–video dataset; public validation dataset with child_id mapping	No known overlap
5	Grossi et al. (32)	Italy	20/40	40 participants (20 ASD, 20 neuropsychiatric controls)	Participant/EEG feature vector	Pediatric	Diagnosis/detection	EEG	Other/Unclear	Other/Unclear	Reviewer–selected best–performing	Clearly defined	Texas archive + prior Italian dataset	No known overlap
6	Li et al. (33)	China	136/272	272 children (136 ASD, 136 TD)	Participant/video (1 per child)	Pediatric	Diagnosis/detection	Eye–tracking/video–audio	RNN/LSTM	Cross–validation	Author–designated primary/final	Clearly defined	Self–collected school–based raw–video dataset	No known overlap
7	Bajestani et al. (34)	Iran	30/60	60 participants (30 ASD, 30 controls)	Participant; 1 selected EEG segment per participant	Pediatric	Diagnosis/detection	EEG	KNN	Cross–validation	Author–designated primary/final	Unclear/insufficient	Self–collected EEG dataset	No known overlap
8	Zhao et al. (35)	China	20/43	43 participants (20 ASD, 23 TD)	Participant; repeated motor trials aggregated within participant	Pediatric	Diagnosis/detection	Clinical/Behavioral	KNN	Cross–validation	Reviewer–selected best–performing	Clearly defined	Self–collected Shenzhen pediatric cohort; restricted–kinematic motor task	Probable overlap: Zhao series
9	Alcañiz Raya (36)	Spain	10/20	92 participants across study phases; evaluation subset *n*=20	Participant	Pediatric	Diagnosis/detection	Multimodal	Support Vector Machine	Hold–out validation	Author–designated primary/final	Clearly defined	Self–collected electrodermal–activity + virtual–reality cohort	No known overlap
10	Drimalla et al. (37)	Germany	36/79	80 main–study participants; evaluation subset *n*=79	Participant; video/audio features aggregated per participant	Adult/Mixed	Diagnosis/detection	Multimodal	Random forest	Cross–validation	Author–designated primary/final	Clearly defined	Original simulated–interaction–task cohort	No known overlap
11	Lai et al. (38)	China	23/46	70 participants overall; matched evaluation cohort *n*=46 (23 ASD, 23 controls)	Participant/retinal–image classification	Pediatric	Diagnosis/detection	Imaging	Support Vector Machine	Cross–validation	Single eligible model	Clearly defined	Self–collected retinal–image cohort	No known overlap
12	Pham et al. (39)	Singapore	40/77	77 participants (40 ASD, 37 controls)	Participant; EEG segments used only for feature processing	Pediatric	Diagnosis/detection	EEG	Other/Unclear	Cross–validation	Author–designated primary/final	Unclear/insufficient	Self–collected EEG cohort	No known overlap
13	Megerian et al. (40)	USA	64/135	425 completers; evaluation subset *n*=135	Participant/child	Pediatric	Diagnosis/detection	Multimodal	Decision Tree	External validation	Single eligible model	Clearly defined	14–site prospective cohort	No known overlap
14	Cheng et al. (41)	China	71/95	95 participants (71 ASD, 24 TD)	Participant; multimodal behavior signals aggregated per child	Pediatric	Diagnosis/detection	Multimodal	Support Vector Machine	Cross–validation	Author–designated primary/final	Clearly defined	Self–collected multimodal behavior dataset	No known overlap
15	Hendr et al. (42)	Cyprus	10/21	104 participants; evaluation subset *n*=21	Participant; 18 handwriting tasks aggregated to one diagnosis	Pediatric	Diagnosis/detection	Clinical/Behavioral	CNN	Cross–validation	Author–designated primary/final	Unclear/insufficient	Author–collected handwriting/drawing dataset	No known overlap
16	Thapa et al. (43)	USA	2,795/7,393	36,965 participants in SPARK primary cohort; evaluation subset *n*=7,393	Participant; four–class output folded to ASD–spectrum vs non–spectrum	Pediatric	Diagnosis/detection	Clinical/Behavioral	Ensemble	Hold–out validation	Single eligible model	Unclear/insufficient	SPARK primary dataset; ABIDE supplementary dataset	Potential overlap: SPARK
17	Wadhera (44)	India	6/12	60 participants (30 ASD, 30 TD); evaluation subset *n*=12	Participant	Unclear	Diagnosis/detection	Multimodal	Support Vector Machine	Hold–out validation	Author–designated primary/final	Unclear/insufficient	Self–collected multimodal EEG + eye–tracking + behavioral dataset	No known overlap
18	Zhao et al. (45)	China	19/39	39 children (19 ASD, 20 TD)	Child–level classification; gaze–behavior segments aggregated to child level	Pediatric	Diagnosis/detection	Eye–tracking/video–audio	Support Vector Machine	Cross–validation	Author–designated primary/final	Clearly defined	Shared Shenzhen pediatric face–to–face conversation cohort; eye–tracking/oculomotor data	Shared cohort: Zhao series
19	Leroy et al. (46)	USA	18/35	185 Arizona ADDM records; evaluation subset *n*=35	Child/case level; sentence predictions aggregated to one child result	Pediatric	Diagnosis/detection	Clinical/Behavioral	Ensemble	Hold–out validation	Author–designated primary/final	Clearly defined	Arizona ADDM records (2014, 2016)	No known overlap
20	Luo et al. (47)	China	20/45	45 participants (20 ASD, 25 controls)	Participant; EEG windows used for feature extraction	Pediatric	Diagnosis/detection	EEG	Support Vector Machine	Cross–validation	Author–designated primary/final	Clearly defined	Institutional EEG cohort	No known overlap
21	Natraj et al. (48)	Switzerland	40/80	160 children (80 ASD, 80 TD); evaluation subset *n*=80	Participant/video; segment predictions aggregated to subject level	Pediatric	Diagnosis/detection	Multimodal	Ensemble	Hold–out validation	Author–designated primary/final	Clearly defined	Self–collected video–audio cohort	No known overlap
22	Xie et al. (49)	China	20/39	39 participants (20 ASD, 19 TD)	Participant; multiple images per subject used for feature learning	Adult/Mixed	Diagnosis/detection	Eye–tracking/video–audio	CNN	Cross–validation	Single eligible model	Clearly defined	Previously established self–collected eye–tracking/visual–attention dataset	No known overlap
23	Perochon et al. (50)	USA	49/475	475 children (49 ASD, 426 non–ASD)	Participant/child; video/gaze features aggregated	Pediatric	Diagnosis/detection	Multimodal	Ensemble	Cross–validation	Author–designated primary/final	Unclear/insufficient	Prospective clinical digital–behavioral–phenotyping cohort	No known overlap
24	Rajagopalan et al. (27)	Sweden	10,476/11,936	SPARK v8 development ≈30,660; external test *n*=11,936 newly recruited SPARK v10 participants	Participant	Adult/Mixed	Diagnosis/detection	Clinical/Behavioral	Ensemble	External validation	Author–designated primary/final	Unclear/insufficient	SPARK v8 development; SPARK v10 new recruits for external test; additional SSC validation	Potential overlap: SPARK
25	Ben–Sasson et al. (51)	Israel	1,164/775,941	Source EHR cohort *n*=780,610; analyzed population *n*=775,941 (1,164 ASD, 774,777 non–ASD)	Participant/longitudinal child EHR record	Pediatric	Diagnosis/detection	Clinical/Behavioral	Ensemble	Cross–validation	Author–designated primary/final	Unclear/insufficient	Maccabi Healthcare Services well–baby EHR	No known overlap
26	Zhao et al. (28)	China	20/41	41 children (20 ASD, 21 TD)	Participant; whole–body movement features aggregated per child	Pediatric	Diagnosis/detection	Eye–tracking/video–audio	Support Vector Machine	Cross–validation	Author–designated primary/final	Clearly defined	Shared Shenzhen pediatric face–to–face conversation cohort; whole–body video data	Shared cohort: Zhao series

ASD, autism spectrum disorder; CNN, convolutional neural network; EEG, electroencephalography; KNN, k–nearest neighbors; RNN/LSTM, recurrent neural network/long short–term memory; TD, typically developing; ADOS, Autism Diagnostic Observation Schedule; ADDM, Autism and Developmental Disabilities Monitoring. The participant-level 2 × 2 analytic sample is shown as ASD cases/total, calculated as TP + FN and TP + FN + FP + TN, respectively; the denominator therefore represents the participant-level evaluation N used in the meta-analysis. The participant-level cohort/sample-size column records the independent participant cohort reported for each study and may exceed the analytic sample when only a subset contributed to the selected 2 × 2 estimate. Unit of analysis specifies the level at which diagnostic classification was evaluated. Dataset source and overlap flag document potential non-independence across reports. ‘Potential overlap' denotes a dataset-level overlap concern without proof of duplicate participants; ‘Shared cohort' indicates clear reuse of the same underlying participant cohort; and ‘Probable overlap' indicates strong evidence of participant overlap without participant identifiers allowing exact matching. For the conservative dataset-independence sensitivity analysis, one representative estimate was retained per overlap cluster [ADOS repositories: Duda et al. (26); SPARK: Rajagopalan et al. (27); Zhao series: Zhao et al. (35)], yielding 21 studies. Model-selection strategy was classified as author-designated primary/final when the original authors prespecified or explicitly selected the main model/configuration for subsequent validation or reporting; reviewer-selected best-performing when multiple eligible models/configurations remained without a unique study-level main model and the review selected the best-performing eligible result; or single eligible model when only one model met the quantitative-synthesis criteria. Reference standard was classified as clearly defined or unclear/insufficient according to the review criteria used for the corresponding sensitivity analysis.

### 2.6 Risk of bias and applicability assessment

Risk of bias and applicability concerns were assessed independently by two reviewers (QW and QHW) using a modified Quality Assessment of Diagnostic Accuracy Studies–Artificial Intelligence (QUADAS-AI) tool (25). Disagreements were resolved through discussion, with arbitration by a third reviewer (RL) when necessary. Although PROBAST was specified in the PROSPERO registration, a modified QUADAS-AI tool was adopted during review conduct because it was considered more appropriate for the diagnostic-accuracy focus of this review and allowed assessment of AI-specific methodological and applicability concerns. This change represented a deviation from the registered protocol.

The assessment covered four domains: patient selection, index test, reference standard, and flow and timing. Patient selection was evaluated with respect to the source and selection of participants, eligibility criteria, sampling approach, use of case-control designs, and the relevance and representativeness of the study population and setting to the review question. The index test domain considered the validation strategy, particularly whether external validation was performed, as well as data preprocessing and partitioning and whether model evaluation was adequately separated from model development, and whether model implementation and interpretation were appropriate for the review question. The reference standard domain addressed whether ASD and non-ASD status were established using an adequately defined diagnostic reference, while flow and timing concerned the interval between the index test and the reference standard. Overall judgments were made on the basis of the signaling questions within each domain. Applicability concerns were assessed separately for patient selection, index test, and reference standard. The modified QUADAS-AI tool was used to assess conventional sources of diagnostic-accuracy bias as well as AI-specific concerns related to data partitioning, model validation, implementation, and clinical applicability.

For the reference-standard sensitivity analysis, the reference standard was considered clearly defined when ASD status was established using recognized diagnostic criteria (e.g., DSM or ICD), standardized autism-specific diagnostic instruments (e.g., ADOS or ADI-R), diagnosis or confirmation by qualified clinicians, or an equivalent clearly documented diagnostic ascertainment procedure. Studies for which the basis of ASD case or control status was insufficiently described or could not be reliably established were classified as having an unclear or insufficiently defined reference standard. This classification was applied specifically to the sensitivity analysis and was separate from the overall QUADAS-AI risk-of-bias judgment for the reference-standard domain.

### 2.7 Statistical analysis

Statistical analyses were performed using R version 4.3.2. Pooled sensitivity and specificity with 95% confidence intervals (CIs) were estimated using a bivariate Reitsma random-effects model fitted by restricted maximum likelihood, accounting for the correlation between the two measures. The random-effects framework was used to accommodate anticipated clinical and methodological heterogeneity across study populations, data modalities, algorithms, and validation strategies. Overall diagnostic performance was summarized using hierarchical summary receiver operating characteristic (HSROC) curves and corresponding AUC values. A continuity correction of 0.5 was applied to zero-cell studies when required.

The primary quantitative synthesis included one participant- or case-level diagnostic-accuracy estimate from each of 26 included studies, selected according to the threshold and model-selection rules described above. To assess the potential influence of non-independent datasets, a conservative dataset-independent sensitivity analysis was performed by retaining one estimate per identified overlap cluster, yielding 21 studies. The representative estimate retained from each identified overlap cluster is documented in Table 1. For the ADOS-repository cluster, Duda et al. (26) was retained because it represented a large subsequent validation sample drawn from multiple ADOS repositories. For the SPARK cluster, Rajagopalan et al. (27) was retained because its principal evaluation used a clearly defined external test cohort of newly recruited SPARK v10 participants. For the Shenzhen/Zhao-series cluster, Zhao et al. (35) was retained because it was the earliest eligible report in the series, used the complete 43-participant cohort, and provided participant-level cross-validation. To assess potential optimism associated with model selection, studies were classified as author-designated primary/final models, reviewer-selected best-performing models, or single eligible models, as described above. Because only three studies contributed reviewer-selected best-performing models, a formal bivariate comparison between model-selection groups was not performed. Sensitivity analyses were performed by excluding the three studies with reviewer-selected best-performing models and, separately, by restricting the analysis to studies with clearly defined reference standards. The overlap between reviewer-selected best-performing models and unclear or inadequate reference standards was examined descriptively.

Subgroup analyses were conducted by age group, validation strategy, data modality, and algorithm family. In addition to the quantitative subgroup analyses, a structured comparative narrative synthesis was conducted to examine differences in study population, data modality, algorithm family, validation strategy, use of external validation, and reported diagnostic performance. Because these methodological characteristics were interrelated and several subgroups included only a small number of studies, the comparisons were interpreted descriptively and not as evidence of independent effects attributable to individual methodological features. For each subgroup, pooled sensitivity and specificity were estimated using the same bivariate framework. HSROC AUCs for the overall analysis, subgroup analyses, and sensitivity analyses were accompanied by bias-corrected and accelerated (BCa) 95% CIs based on 5,000 study-level bootstrap replicates. Point AUC estimates were calculated from the Rutter–Gatsonis SROC curve derived from the original fitted Reitsma model using the AUC.reitsma method in the mada package. AUCs were not reported when the fitted SROC curve was considered unstable. For each bootstrap replicate, studies were resampled with replacement, the bivariate Reitsma model was refitted, and the HSROC AUC was recalculated using the same Rutter–Gatsonis SROC-based procedure as for the point estimate; the acceleration parameter for the BCa interval was estimated using leave-one-study-out jackknife resampling. For subgroup analyses, AUCs were not reported when the original fitted Reitsma model showed an unstable random-effects covariance structure, and BCa confidence intervals were not calculated in those cases. To avoid unstable pooled estimates from extremely sparse subgroups, subgroup-specific pooled estimates were not calculated for categories containing fewer than four studies; these categories were summarized descriptively.

Between-study heterogeneity in sensitivity and specificity was summarized using I^2^, τ^2^, and Cochran's Q *P* values. Potential sources of heterogeneity were examined using separate univariable bivariate random-effects meta-regression models within the Reitsma framework for age group, data modality, validation strategy, and broad algorithm class. Overall covariate effects were assessed by likelihood-ratio tests comparing maximum-likelihood models with and without the corresponding covariate, fitted to the same subset of studies. Coefficients and variance components were estimated using restricted maximum likelihood. Group-specific sensitivity and specificity estimates and 95% CIs were calculated as fixed-effect linear combinations from the full REML meta-regression models using the complete fixed-effect covariance matrix and back-transformed to the probability scale. Studies with unclear classifications or extremely sparse categories were excluded only from the corresponding meta-regression. Specifically, the single imaging study was excluded from the modality meta-regression but retained in the descriptive subgroup analysis. For the broad algorithm-class meta-regression, individual algorithm families were grouped into three categories: traditional non-ensemble machine-learning methods, ensemble methods, and deep neural networks. Changes in estimated random-effects variances relative to the corresponding intercept-only models were summarized descriptively. Metric-specific Wald tests and pairwise odds-ratio contrasts were considered exploratory secondary analyses and were not interpreted as diagnostic odds ratios. Multivariable and interaction models were not fitted because several cross-classified cells contained too few studies to permit stable estimation.

The primary synthesis was further assessed using leave-one-out sensitivity analysis. Each study was sequentially omitted, and the bivariate Reitsma random-effects model was refitted using the remaining 25 studies. The resulting pooled sensitivity and specificity estimates and their 95% CIs were compared with those obtained from the complete 26-study dataset. For each metric, the range of leave-one-out estimates and the largest absolute change from the full-data estimate were recorded.

Funnel-plot asymmetry was assessed using Deeks' test. The test was applied to the primary participant-level dataset (*n* = 26), the dataset-independent sensitivity dataset (*n* = 21), the dataset excluding reviewer-selected best-performing models (*n* = 23), and the dataset restricted to studies with clearly defined reference standards (*n* = 16). The direction of the regression slope was reported together with its statistical significance. A two-sided *P* < 0.05 was considered to indicate significant funnel-plot asymmetry.

## 3 Results

### 3.1 Study selection

A total of 17,387 records were identified through database searches, including PubMed (*n* = 3,220), Embase (*n* = 3,005), the Cochrane Library (*n* = 180), Web of Science (*n* = 8,330), and IEEE Xplore (*n* = 2,652). Before screening, 175 duplicate records were removed. The remaining 17,212 records underwent title and abstract screening, of which 17,110 were excluded.

The full texts of 102 reports were sought and successfully retrieved. All 102 reports were assessed for eligibility, of which 76 were excluded for the following reasons: outside the scope of the review (*n* = 28), insufficient diagnostic accuracy data to extract or reconstruct a 2 × 2 contingency table (*n* = 22), overlapping data (*n* = 7), unpublished conference abstracts (*n* = 5), diagnostic counts based on lower-level observations such as images, EEG segments, sessions, or raw samples (*n* = 6), inability to reliably reconstruct a unique participant-level 2 × 2 table from the reported validation results (*n* = 7), and a surveillance case-ascertainment task that did not meet the clinically oriented task criterion (*n* = 1). Consequently, 26 studies were included in the systematic review and primary quantitative synthesis. The study selection process is shown in Figure 1.

**Figure 1:**
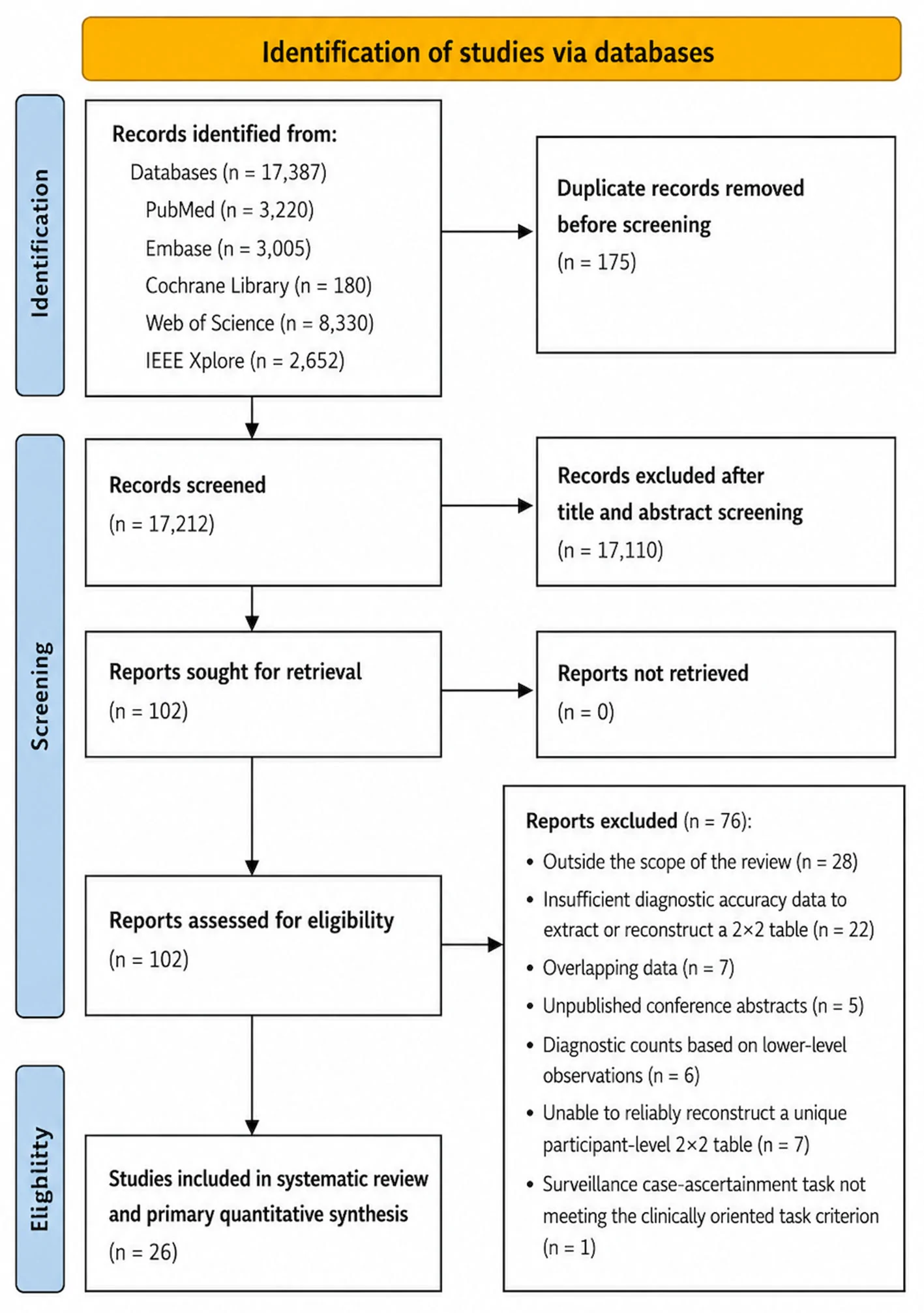
Flow diagram of the study selection process.

### 3.2 Study characteristics

The main characteristics of the 26 studies included in the systematic review and meta-analysis are summarized in Table 1 (26–51). The studies were published between 2012 and 2025 and were conducted across multiple countries, with China (*n* = 8) and the United States (*n* = 8) contributing the largest numbers. One eligible diagnostic-accuracy estimate for binary ASD-versus-non-ASD classification was retained from each study. All 2 × 2 tables used in the primary analysis represented participant- or case-level classifications rather than image-, segment-, or trial-level observations. Where lower-level images, signal segments, repeated trials, or video segments were used for feature extraction or intermediate prediction, the retained diagnostic result was aggregated or evaluated at the participant level. Participant-level cohort size and the number of participants contributing to the selected 2 × 2 table are reported separately in Table 1 when these differed.

Regarding data modality, nine studies used clinical or behavioral data, five used eye-tracking or video/audio data, four used EEG data, one used imaging data, and seven used multimodal data. Most studies focused on pediatric populations: 21 included children or adolescents, four involved adult or mixed-age samples, and one had an unclear age classification. The included studies used a range of AI algorithms. Support vector machines were the most common (*n* = 8), followed by ensemble methods (*n* = 6), decision trees (*n* = 3), CNNs (*n* = 2), KNNs (*n* = 2), other or unclear algorithms (*n* = 2), random forests (*n* = 1), RNN/LSTM models (*n* = 1), and logistic regression (*n* = 1).

Regarding validation strategies, 15 studies used cross-validation, five used hold-out validation, five contributed externally validated diagnostic-accuracy estimates, and one used an other or unclear validation approach. Eighteen studies contributed estimates from models designated by the original authors as the primary, final, or main model, three contributed reviewer-selected best-performing models, and five had only one eligible model. Sixteen studies had clearly defined reference standards according to the operational criteria used for the reference-standard sensitivity analysis, whereas ten were classified as unclear or insufficiently defined.

Potential non-independence of data sources was identified across three groups of reports: three studies drew on overlapping ADOS repositories, two used SPARK data, and three were based on the same or probably overlapping Shenzhen cohort. These relationships are documented in Table 1. A conservative dataset-independent sensitivity analysis retaining one estimate per overlap cluster was therefore based on 21 studies. Overall, the evidence base remained predominantly pediatric and internally validated, with relatively limited evaluation in independent external datasets.

### 3.3 Risk of bias and applicability concerns

Risk of bias and applicability concerns were assessed across the 26 included studies using the modified QUADAS-AI tool. Risk of bias was evaluated across four domains: patient selection, index test, reference standard, and flow and timing. Applicability concerns were evaluated across three domains: patient selection, index test, and reference standard. The summary of judgments is presented in Figure 2.

**Figure 2:**
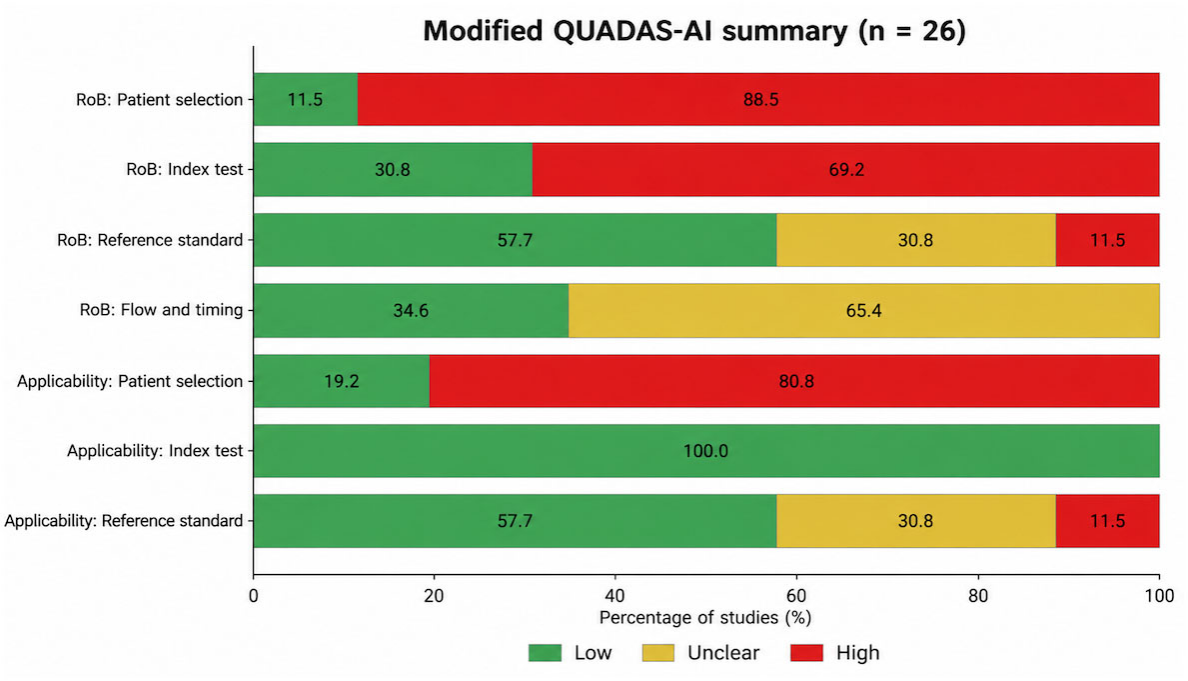
Summary of risk of bias and applicability concerns for the included studies assessed using the modified QUADAS-AI tool.

#### 3.3.1 Risk of bias

Methodological concerns were most frequent in the patient selection and index test domains. In the patient selection domain, 23 studies were rated as high risk (88.5%) and three as low risk (11.5%). In the index test domain, 18 studies were rated as high risk (69.2%) and eight as low risk (30.8%). High-risk judgments in these domains were mainly related to selected or convenience samples, case-control designs, narrowly defined study populations, and the lack of independent model evaluation.

In the reference standard domain, three studies were rated as high risk (11.5%), eight as unclear risk (30.8%), and 15 as low risk (57.7%). Unclear or high-risk judgments were mainly related to incomplete reporting of the diagnostic procedure or insufficiently defined non-ASD reference labels. In the flow and timing domain, 17 studies were rated as unclear risk (65.4%) and nine as low risk (34.6%), with no study rated as high risk. The unclear judgments were mainly related to insufficient reporting of the interval between the reference diagnosis and the AI-based assessment (Figure 2).

#### 3.3.2 Applicability concerns

Applicability concerns were mainly related to patient selection. In the patient selection domain, 21 studies were rated as high concern (80.8%) and five as low concern (19.2%), with no study rated as unclear. The high applicability concerns were largely related to the use of selected or convenience samples, case-control designs, narrowly defined study populations, and comparison groups that may not fully represent the broader clinical populations in which ASD detection models would ultimately be applied.

Applicability concerns were minimal in the index test domain, with all 26 studies rated as low concern (100.0%). In the reference standard domain, three studies were rated as high concern (11.5%), eight as unclear concern (30.8%), and 15 as low concern (57.7%). Unclear or high applicability concerns were mainly associated with insufficiently documented diagnostic procedures or reference labels that did not fully correspond to a clearly established clinical diagnosis (Figure 2).

#### 3.3.3 Overall assessment

Overall, the modified QUADAS-AI assessment identified patient selection and model evaluation as the main methodological limitations of the available evidence, with additional concerns related to incomplete reporting of reference standards and flow and timing.

### 3.4 Overall diagnostic performance

In the primary participant-level analysis of 26 studies using the bivariate Reitsma random-effects model, pooled sensitivity was 0.908 (95% CI: 0.861–0.940; Figure 3A) and pooled specificity was 0.868 (95% CI: 0.830–0.898; Figure 3B). The corresponding HSROC AUC was 0.936 (BCa 95% CI: 0.860–0.973; Figure 4). Substantial heterogeneity was observed for both sensitivity (*I*^2^ = 96.7%; τ^2^ = 1.05; *P* < 0.001) and specificity (*I*^2^ = 95.8%; τ^2^ = 0.30; *P* < 0.001).

**Figure 3:**
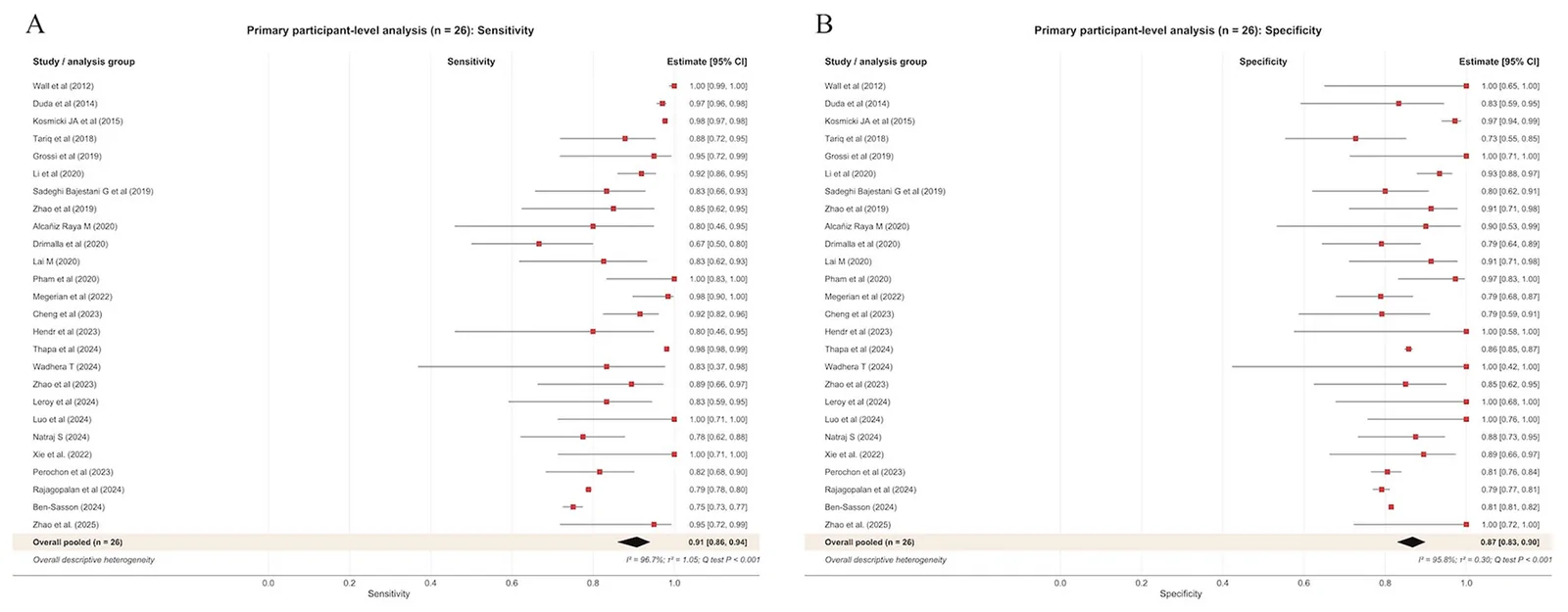
Forest plots of overall diagnostic performance of AI models for ASD detection: **(A)** sensitivity and **(B)** specificity. Pooled estimates and 95% confidence intervals are shown together with study-specific estimates.

**Figure 4:**
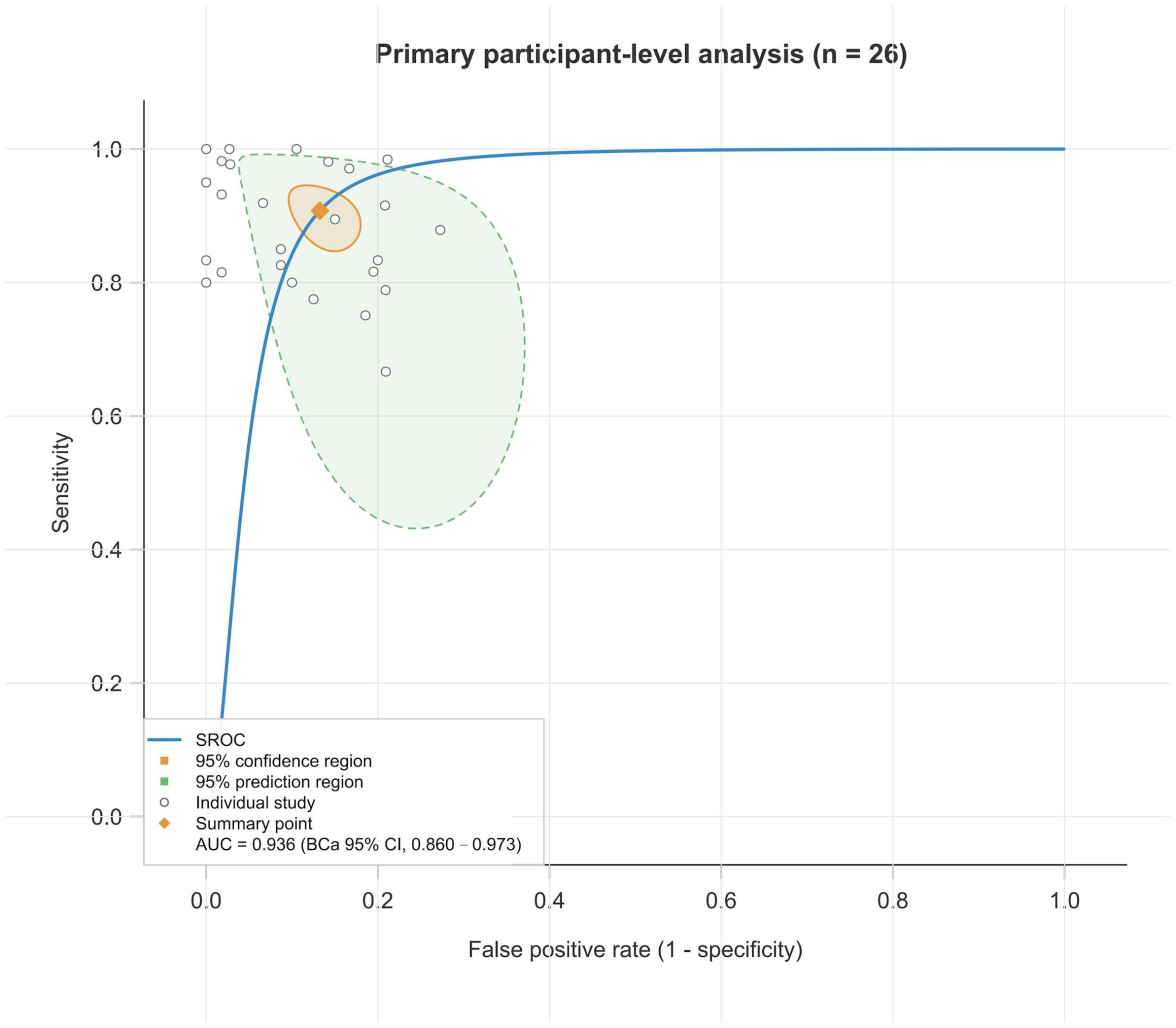
HSROC curve of overall diagnostic performance in the primary participant-level analysis (*n* = 26). The summary point, 95% confidence region, 95% prediction region, and HSROC AUC with BCa 95% confidence interval are shown.

### 3.5 Sensitivity analyses related to dataset overlap, model selection, and reference standards

To examine the potential influence of non-independent datasets, the analysis was repeated after retaining one representative estimate per identified overlap cluster. This dataset-independent analysis included 21 studies and yielded a pooled sensitivity of 0.870 (95% CI: 0.820–0.908), pooled specificity of 0.841 (95% CI: 0.813–0.865), and an HSROC AUC of 0.900 (BCa 95% CI: 0.813–0.956; Figure 5). Compared with the primary 26-study analysis, pooled sensitivity decreased from 0.908 to 0.870, pooled specificity from 0.868 to 0.841, and the HSROC AUC from 0.936 to 0.900.

**Figure 5:**
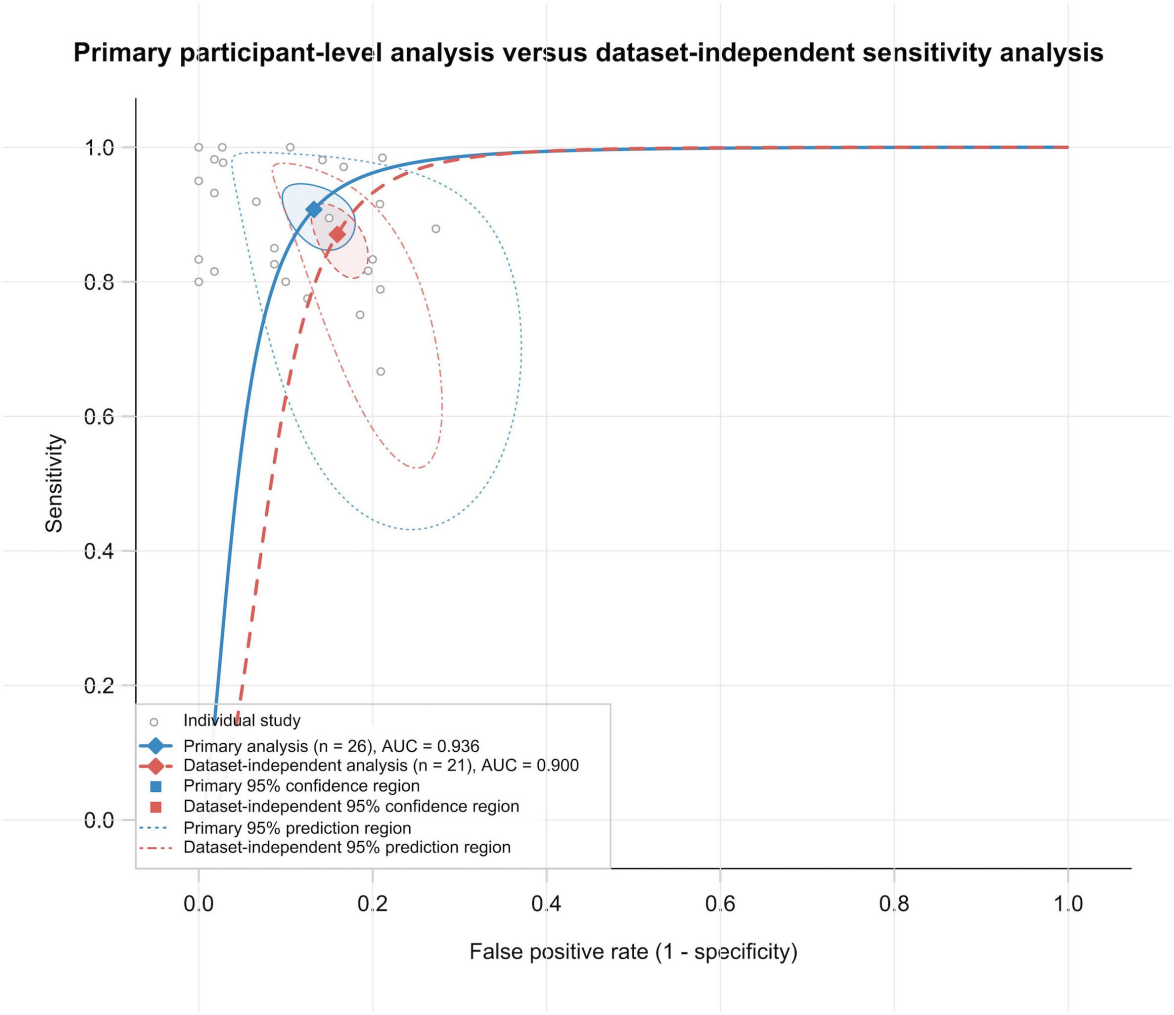
HSROC curves comparing the primary participant-level analysis (*n* = 26) and the dataset-independent sensitivity analysis (*n* = 21). Summary points, 95% confidence regions, and 95% prediction regions are shown for both analyses.

Among the 26 studies in the primary analysis, 18 contributed estimates from models designated by the original authors as primary, final, or main models, three contributed reviewer-selected best-performing models because no primary or final model had been specified, and five reported a single eligible model. Given the small number of studies in the reviewer-selected group, a formal bivariate comparison between model-selection strategies was not performed. After excluding the three studies with reviewer-selected best-performing models, the remaining 23 studies had a pooled sensitivity of 0.901 (95% CI: 0.848–0.937), pooled specificity of 0.829 (95% CI: 0.814–0.844), and an HSROC AUC of 0.867 (BCa 95% CI: 0.804–0.936; Supplementary Figure S1). Relative to the primary analysis, pooled sensitivity decreased from 0.908 to 0.901, pooled specificity from 0.868 to 0.829, and the HSROC AUC from 0.936 to 0.867, with the largest attenuation observed for AUC.

When the analysis was restricted to the 16 studies with clearly defined reference standards, pooled sensitivity was 0.910 (95% CI: 0.852–0.946), pooled specificity was 0.903 (95% CI: 0.854–0.937), and the HSROC AUC was 0.957 (BCa 95% CI: 0.911–0.980; Supplementary Figure S2). Compared with the primary analysis, sensitivity, specificity, and AUC changed by +0.002, +0.035, and +0.021, respectively. Restricting the synthesis to studies with clearly defined reference standards therefore did not reduce the pooled diagnostic-performance estimates.

### 3.6 Subgroup analyses

Subgroup analyses were performed according to age group, validation strategy, data modality, and algorithm family. Pooled sensitivity and specificity varied numerically across the estimable subgroups, and these differences were further examined using meta-regression. HSROC AUCs were reported only for subgroups in which the SROC fit was considered sufficiently stable; subgroups with fewer than four studies were described rather than pooled. The subgroup results are summarized in Table 2, and forest plots of pooled sensitivity and specificity are provided in Supplementary Figures S3–S6.

**Table 2:** Subgroup analyses of pooled diagnostic performance of artificial intelligence models for autism spectrum disorder detection.

Subgroup domain	Subgroup	Studies, *n*	Pooled sensitivity (95% CI)	Pooled specificity (95% CI)	HSROC AUC (BCa 95% CI)
Age group	Pediatric	21	0.911 (0.865–0.942)	0.875 (0.833–0.908)	0.945 (0.859–0.973)
	Adult/Mixed	4	0.953 (0.556–0.997)	0.858 (0.737–0.928)	Not reported^*^
	Unclear	1	Not pooled	Not pooled	Not pooled
Validation strategy	Cross–validation	15	0.888 (0.820–0.932)	0.884 (0.847–0.914)	Not reported^*^
	Hold–out validation	5	0.878 (0.688–0.959)	0.886 (0.867–0.903)	Not reported^*^
	External validation	5	0.940 (0.848–0.978)	0.856 (0.712–0.934)	0.956 (0.792–0.982)
	Other/Unclear	1	Not pooled	Not pooled	Not pooled
Data modality	Clinical/Behavioral	9	0.934 (0.833–0.976)	0.889 (0.815–0.935)	0.953 (0.858–0.986)
	Eye–tracking/Video–Audio	5	0.910 (0.860–0.944)	0.878 (0.765–0.941)	Not reported^*^
	EEG	4	0.939 (0.821–0.981)	0.937 (0.801–0.982)	Not reported^*^
	Multimodal	7	0.835 (0.724–0.906)	0.807 (0.773–0.837)	Not reported^*^
	Imaging	1	Not pooled	Not pooled	Not pooled
Algorithm family	Support Vector Machine	8	0.922 (0.854–0.959)	0.909 (0.828–0.954)	Not reported^*^
	Ensemble	6	0.857 (0.707–0.937)	0.820 (0.798–0.840)	0.850 (0.746–0.866)
	Decision Tree	3	Not pooled	Not pooled	Not pooled
	CNN	2	Not pooled	Not pooled	Not pooled
	KNN	2	Not pooled	Not pooled	Not pooled
	Other/Unclear	2	Not pooled	Not pooled	Not pooled
	Random Forest	1	Not pooled	Not pooled	Not pooled
	RNN/LSTM	1	Not pooled	Not pooled	Not pooled
	Logistic Regression	1	Not pooled	Not pooled	Not pooled

Pooled sensitivity and specificity are presented as proportions with 95% confidence intervals and rounded to three decimals; HSROC AUC values are presented with BCa 95% confidence intervals and rounded to three decimals. Categories containing fewer than four studies were summarized descriptively and were not pooled. ^*^AUC was not reported when the fitted SROC curve or random–effects covariance structure was unstable. AUC, area under the curve; BCa, bias-corrected and accelerated; HSROC, hierarchical summary receiver operating characteristic; EEG, electroencephalography; RNN, recurrent neural network; LSTM, long short-term memory; KNN, k-nearest neighbors; CNN, convolutional neural network.

#### 3.6.1 Age group

The pediatric subgroup included 21 studies, with a pooled sensitivity of 0.911 (95% CI: 0.865–0.942) and a pooled specificity of 0.875 (95% CI: 0.833–0.908; Supplementary Figures S3A, B). The corresponding HSROC AUC was 0.945 (BCa 95% CI: 0.859–0.973).

Four studies were included in the adult/mixed-age subgroup, with a pooled sensitivity of 0.953 (95% CI: 0.556–0.997) and a pooled specificity of 0.858 (95% CI: 0.737–0.928). The wide confidence interval for sensitivity reflected the small number of studies in this subgroup. Because the fitted SROC was unstable, an AUC was not reported for the adult/mixed-age subgroup. One study was classified in the unclear-age subgroup and was therefore described without pooling. Meta-regression showed no significant overall association between age group and diagnostic performance (*P* = 0.879; Section 3.7).

#### 3.6.2 Validation strategy

Among studies using cross-validation (*n* = 15), pooled sensitivity was 0.888 (95% CI: 0.820–0.932) and pooled specificity was 0.884 (95% CI: 0.847–0.914; Supplementary Figures S4A, B). For hold-out validation (*n* = 5), the corresponding estimates were 0.878 (95% CI: 0.688–0.959) and 0.886 (95% CI: 0.867–0.903), respectively. Among the five studies contributing externally validated estimates, pooled sensitivity was 0.940 (95% CI: 0.848–0.978) and pooled specificity was 0.856 (95% CI: 0.712–0.934). One study was classified as having an other or unclear validation strategy and was not pooled.

The HSROC AUC for the external-validation subgroup was 0.956 (BCa 95% CI: 0.792–0.982). AUCs were not reported for the cross-validation or hold-out subgroups because their fitted SROC shapes were unstable. Meta-regression showed no significant overall association between validation strategy and diagnostic performance (*P* = 0.181; Section 3.7).

#### 3.6.3 Data modality

Among the nine studies using clinical or behavioral data, pooled sensitivity was 0.934 (95% CI: 0.833–0.976) and pooled specificity was 0.889 (95% CI: 0.815–0.935; Supplementary Figures S5A, B). The corresponding HSROC AUC was 0.953 (BCa 95% CI: 0.858–0.986).

For eye-tracking or video/audio data (*n* = 5), pooled sensitivity was 0.910 (95% CI: 0.860–0.944) and pooled specificity was 0.878 (95% CI: 0.765–0.941). Four studies used EEG data, with a pooled sensitivity of 0.939 (95% CI: 0.821–0.981) and pooled specificity of 0.937 (95% CI: 0.801–0.982). Seven studies used multimodal data, with corresponding estimates of 0.835 (95% CI: 0.724–0.906) and 0.807 (95% CI: 0.773–0.837), respectively. AUCs were not reported for these three subgroups because the fitted SROC shapes were unstable. The imaging subgroup included only one study and was therefore described without pooling. Meta-regression showed no significant overall association between data modality and diagnostic performance (*P* = 0.739; Section 3.7).

#### 3.6.4 Algorithm family

Subgroup analysis by algorithm family showed numerical variation in pooled diagnostic estimates (Supplementary Figures S6A, B). Eight studies used support vector machines, with a pooled sensitivity of 0.922 (95% CI: 0.854–0.959) and pooled specificity of 0.909 (95% CI: 0.828–0.954). Six studies used ensemble methods, with a pooled sensitivity of 0.857 (95% CI: 0.707–0.937) and pooled specificity of 0.820 (95% CI: 0.798–0.840).

The HSROC AUC for the ensemble subgroup was 0.850 (BCa 95% CI: 0.746–0.866). An AUC was not reported for the support vector machine subgroup because the fitted SROC was unstable. No pooled estimates were calculated for decision trees, CNNs, KNNs, logistic regression, random forests, RNN/LSTM, or other/unclear algorithms because each category included fewer than four studies. Broad algorithm class showed a significant overall association with diagnostic performance in meta-regression (*P* = 0.030; Section 3.7), although individual group contrasts were interpreted cautiously.

The unstable fitted SROC curves for the adult/mixed-age, cross-validation, hold-out validation, eye-tracking/video-audio, EEG, multimodal, and support vector machine subgroups are shown in Supplementary Figures S7A–G.

### 3.7 Meta-regression analyses of heterogeneity

Separate univariable bivariate random-effects meta-regression models within the Reitsma framework showed no significant overall association of diagnostic performance with age group (*n* = 25; likelihood-ratio χ^2^ = 0.257, df = 2, *P* = 0.879), data modality (*n* = 25; χ^2^ = 3.535, df = 6, *P* = 0.739), or validation strategy (*n* = 25; χ^2^ = 6.248, df = 4, *P* = 0.181; Figure 6).

**Figure 6:**
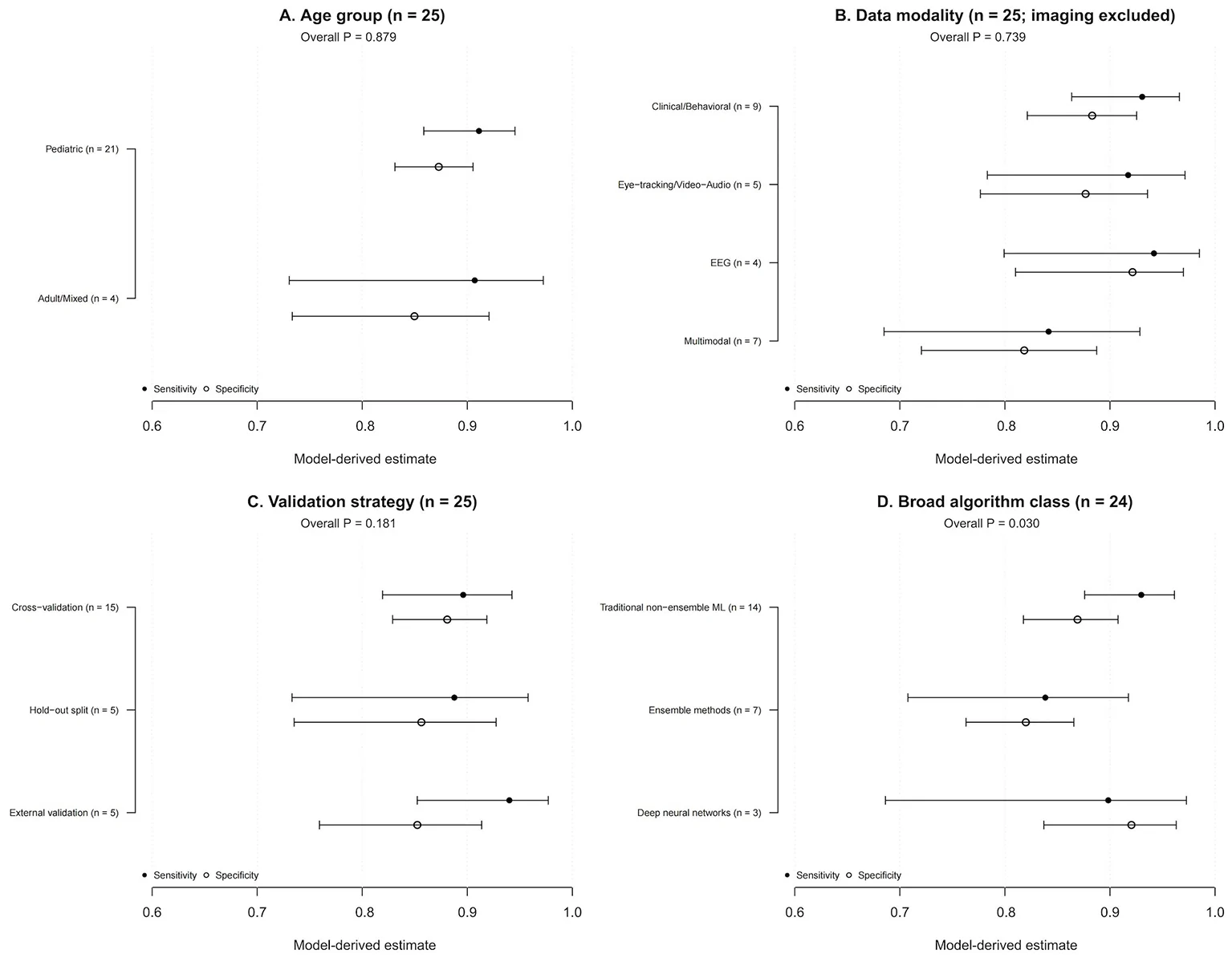
Meta-regression estimates of diagnostic performance according to **(A)** age group, **(B)** data modality, **(C)** validation strategy, and **(D)** broad algorithm class. Points and error bars represent model-derived sensitivity and specificity estimates with 95% confidence intervals; overall *P* values are from likelihood-ratio tests.

Broad algorithm class showed a significant overall association with diagnostic performance (*n* = 24; χ^2^ = 10.687, df = 4, *P* = 0.030). The analysis included 14 studies using traditional non-ensemble machine-learning methods, seven using ensemble methods, and three using deep neural networks; two studies classified as other/unclear were excluded. Model-derived sensitivity and specificity estimates were 0.930 (95% CI: 0.876–0.961) and 0.869 (95% CI: 0.818–0.908), respectively, for traditional non-ensemble machine learning; 0.838 (95% CI: 0.708–0.918) and 0.820 (95% CI: 0.763–0.866) for ensemble methods; and 0.898 (95% CI: 0.686–0.973) and 0.920 (95% CI: 0.837–0.963) for deep neural networks.

However, exploratory metric-specific Wald tests were not statistically significant for sensitivity (*P* = 0.179) or false-positive rate (*P* = 0.078), and none of the pairwise sensitivity or specificity contrasts relative to traditional non-ensemble machine learning reached statistical significance (Supplementary Figure S8). Inclusion of broad algorithm class was associated with descriptive reductions of 7.3% and 35.2% in the estimated random-effects variances on the logit-sensitivity and logit false-positive-rate scales, respectively. The single imaging study was retained in the descriptive subgroup analysis but excluded from the modality meta-regression. Because the deep-neural-network subgroup contained only three studies and several cross-classified cells were sparse, these study-level associations were interpreted cautiously.

### 3.8 Leave-one-out sensitivity analysis

Leave-one-out sensitivity analysis showed that the pooled diagnostic estimates were generally stable after sequential omission of individual studies (Supplementary Figure S9). Across the 26 leave-one-out analyses, pooled sensitivity ranged from 0.894 to 0.913, compared with 0.908 (95% CI: 0.861–0.940) in the primary analysis. The largest absolute change in pooled sensitivity occurred when Thapa et al. (43) was omitted, yielding 0.894 (95% CI: 0.848–0.928), compared with 0.908 in the primary analysis.

Pooled specificity ranged from 0.830 to 0.875, compared with 0.868 (95% CI: 0.830–0.898) in the primary analysis. The largest absolute change occurred when Kosmicki et al. (30) was omitted, yielding a pooled specificity of 0.830 (95% CI: 0.815–0.844), an absolute change of 0.038. Overall, the pooled estimates were relatively stable to the omission of individual studies, although specificity showed greater sensitivity to individual-study omission than sensitivity.

### 3.9 Funnel-plot asymmetry

Deeks' test showed no significant funnel-plot asymmetry in the primary 26-study participant-level analysis (slope = 7.820, t = 0.678, df = 24, *P* = 0.504; Figure 7A). The slope was positive, indicating that smaller studies tended to have higher diagnostic odds ratios (DORs), although this pattern was not statistically significant.

**Figure 7:**
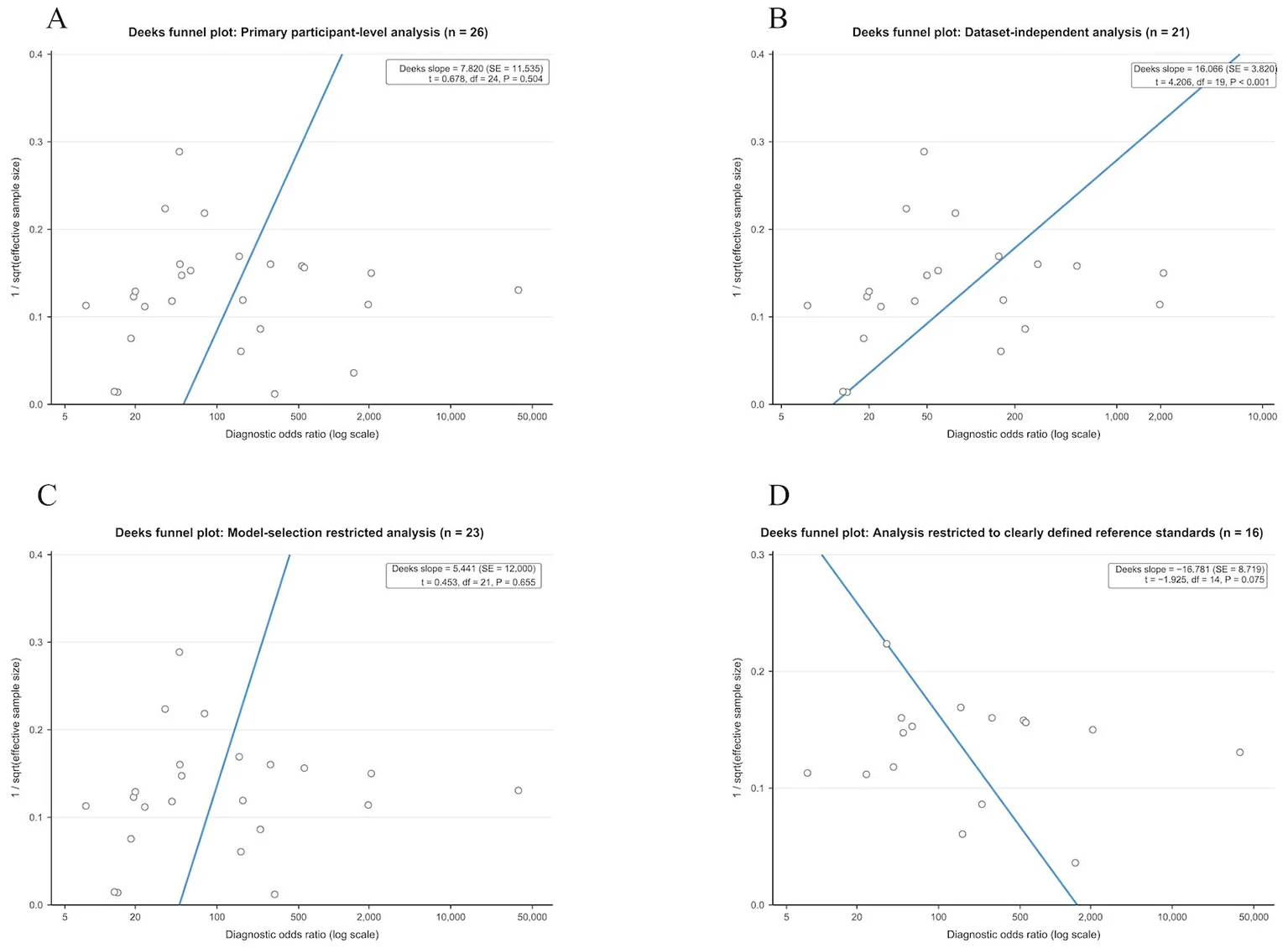
Deeks' funnel plots for **(A)** the primary participant-level analysis, **(B)** the dataset-independent analysis, **(C)** the analysis excluding reviewer-selected best-performing models, and **(D)** the analysis restricted to studies with clearly defined reference standards. Regression slopes and corresponding *P* values are shown in each panel.

In the dataset-independent analysis of 21 studies, the slope remained positive and funnel-plot asymmetry was statistically significant (slope = 16.066, *t* = 4.206, df = 19, *P* < 0.001; Figure 7B), with smaller studies tending to report higher DORs. No significant asymmetry was observed after excluding the three studies with reviewer-selected best-performing models (*n* = 23; slope = 5.441, *t* = 0.453, df = 21, *P* = 0.655; Figure 7C), although the slope remained positive.

Among the 16 studies with clearly defined reference standards, the slope was negative (slope = −16.781, t = −1.925, df = 14, *P* = 0.075; Figure 7D), indicating that smaller studies tended to have lower rather than higher DORs. Overall, the direction and statistical significance of funnel-plot asymmetry varied across the different analysis sets, providing no consistent evidence of conventional small-study effects across analyses.

## 4 Discussion

The primary participant-level meta-analysis of AI-assisted approaches for ASD detection suggested relatively high diagnostic performance in research settings. These findings, however, should not be interpreted as evidence of consistent performance across clinical settings. Between-study heterogeneity remained very high, external validation was uncommon, and concerns remained regarding risk of bias, applicability, model selection, the quality and reporting of reference standards, and potential non-independence of underlying datasets. Sensitivity analyses showed some attenuation of pooled performance after retaining one estimate per overlapping dataset and after excluding reviewer-selected best-performing models, whereas restricting the analysis to studies with clearly defined reference standards did not reduce the diagnostic-performance estimates. Funnel-plot asymmetry was not significant in the primary participant-level analysis and varied in both direction and statistical significance across sensitivity analyses, precluding a consistent interpretation as evidence of conventional small-study effects or selective reporting. In addition, the evidence base was dominated by pediatric populations and internally validated models, limiting confidence in generalizability to broader clinical populations and independent settings. Taken together, the available evidence suggests that AI may have value as an adjunct to established ASD assessment pathways, particularly for screening, referral support, and clinical decision-making, but does not yet support its use as a standalone diagnostic system (52–55).

Pediatric populations dominated the evidence base. However, meta-regression showed no significant association between age group and diagnostic performance, providing no evidence that performance was systematically better in pediatric populations. Although the adult/mixed-age subgroup showed numerically high sensitivity, the estimate was imprecise and the fitted SROC was unstable because few studies were available. Evidence in adult and mixed-age populations therefore remains limited, and population-specific validation is needed before models developed mainly in pediatric samples can be generalized to older populations (53, 54).

Validation strategy is important when considering the clinical relevance and transportability of reported diagnostic performance. Most included studies relied on internal validation, whereas external validation remained limited. Although externally validated models showed numerical differences in sensitivity and specificity, meta-regression did not show a significant association between validation strategy and diagnostic performance, and these differences should not be interpreted as evidence that one validation approach is superior. Instead, the limited external-validation evidence leaves uncertainty about whether model performance can be maintained across independent populations, institutions, acquisition settings, and clinical workflows. Consistent with this concern, a meta-analysis specifically of home-video-based machine-learning approaches reported lower pooled sensitivity and specificity in validation cohorts than in cross-validation analyses, further highlighting the importance of independent validation before clinical translation (56). Future studies should prioritize prospective, multicenter external validation using locked models and prespecified operating thresholds, with reporting guided by TRIPOD+AI and STARD-AI and methodological appraisal supported by PROBAST+AI (57–59).

Diagnostic performance varied numerically across data modalities, with EEG-based studies showing the highest pooled sensitivity and specificity among the estimable modality subgroups, whereas multimodal studies showed numerically lower pooled estimates. However, meta-regression showed no significant association between data modality and diagnostic performance, so these differences should not be interpreted as evidence of inherent superiority of any modality. Stable HSROC AUC estimates could not be obtained for several modality subgroups, further limiting direct comparisons. Observed differences may also reflect variation in participant characteristics, preprocessing, feature extraction, validation strategy, and clinical setting rather than modality alone. The imaging subgroup included only one study and was therefore summarized descriptively. The relatively lower performance of multimodal studies should be interpreted cautiously and may depend on data completeness, fusion strategy, model complexity, and validation design.

These findings should also be considered in relation to previous systematic reviews. A recent systematic review and meta-analysis focusing on AI-based analysis of facial and social-behavioral information for ASD assessment also reported promising diagnostic performance, while emphasizing the need for further validation in diverse populations (60). Compared with the broader review by Valizadeh et al. (61), our review used a more restrictive diagnostic-accuracy framework, requiring participant- or case-level data sufficient to construct or reconstruct a binary ASD-versus-non-ASD 2 × 2 table and retaining one eligible diagnostic estimate per included study. Valizadeh et al. synthesized results by modality using the Split Component Synthesis approach and assessed risk of bias with an adapted QUADAS-2 tool. In contrast, we jointly analyzed sensitivity and specificity using a bivariate Reitsma/HSROC framework and assessed study quality using a modified QUADAS-AI tool. We also evaluated dataset non-independence, model selection, reference-standard quality, heterogeneity, and the influence of individual studies through sensitivity analyses and meta-regression. Together, these methodological features allowed a more focused assessment of the robustness and generalizability of AI-assisted ASD diagnostic performance.

Diagnostic performance varied across algorithm families, and broad algorithm class showed an overall association with diagnostic performance in meta-regression. However, exploratory metric-specific tests and pairwise contrasts did not identify significant differences between individual algorithm classes, and substantial residual heterogeneity remained. Given the small number of deep-neural-network studies and potential confounding by data modality, sample characteristics, and validation design, these findings suggest that algorithm class may contribute to between-study heterogeneity but do not establish the superiority of any specific approach.

Sensitivity analyses provided additional context for interpreting the pooled estimates. Retaining one representative estimate per identified overlap cluster led to modest attenuation of pooled performance, suggesting that dataset overlap may contribute some optimism to study-level estimates. Excluding reviewer-selected best-performing models also reduced performance, particularly for specificity and AUC, although this finding should be interpreted cautiously because few studies required review-level model selection. By contrast, restricting the analysis to studies with clearly defined reference standards did not reduce pooled performance, suggesting that the primary findings were not driven by studies with unclear or insufficiently defined reference standards. Together, these analyses underscore the importance of dataset independence, prespecified or author-designated models, and clearly defined reference standards in future evaluations of AI-assisted ASD detection.

The modified QUADAS-AI assessment identified substantial methodological limitations, particularly in patient selection and index-test evaluation, with additional concerns regarding reference standards and flow and timing. Applicability concerns were concentrated in patient selection, as many studies relied on convenience samples, case-control designs, narrowly defined populations, or comparison groups that may not reflect broader clinical settings. Consequently, performance observed in comparisons between ASD cases and typically developing controls may not generalize to clinically relevant differential-diagnostic populations, particularly when independent model evaluation is lacking. These findings underscore the need for representative sampling, clinically appropriate comparison groups, and independent model evaluation before broader clinical application (25).

Leave-one-out analysis showed that the pooled estimates were generally stable, although specificity was more sensitive to individual-study omission. This numerical stability, however, does not address methodological limitations shared across studies. Funnel-plot asymmetry was not statistically significant in the primary participant-level analysis and varied in both direction and statistical significance across the sensitivity analyses. Although significant positive asymmetry was observed in the dataset-independent analysis, the remaining analyses were not statistically significant, and the reference-standard-restricted analysis showed a negative slope. These inconsistent patterns do not support a consistent interpretation of conventional small-study effects or selective reporting and should therefore be interpreted cautiously.

Several evidence gaps need to be addressed before AI-assisted ASD detection can be considered for routine clinical use. Future studies should enroll representative and clinically heterogeneous populations, including adults, use clearly defined reference diagnoses and clinically relevant comparison groups, and ensure strict participant-level separation of model development, tuning, and testing. Diagnostic performance should be evaluated at the participant or case level rather than treating repeated images, signal segments, or trials from the same individual as independent observations, and evaluation datasets should be independent of model-development data and, where possible, independent of datasets used in previous studies. Prospective multicenter external validation using locked models and prespecified operating thresholds should be prioritized to assess transportability across clinical settings. Evaluation should also extend beyond discrimination to calibration, clinical utility, fairness, interpretability, and implementation feasibility. These priorities are consistent with recent consensus guidance for trustworthy healthcare AI (62).

This review has several strengths, including the use of a bivariate diagnostic-accuracy framework, modified QUADAS-AI, and formal meta-regression. The synthesis was restricted to participant- or case-level diagnostic classifications, with targeted sensitivity analyses addressing dataset non-independence, model selection, and reference-standard quality. HSROC AUC uncertainty was assessed using BCa bootstrap confidence intervals, and AUCs were withheld when fitted SROC curves were unstable. Together, these approaches allowed a more robust assessment of diagnostic performance and the reliability of the underlying evidence.

## 5 Conclusion

AI-assisted approaches show promising diagnostic performance for ASD detection, but the current evidence is not yet sufficiently robust to support broad clinical implementation. Substantial heterogeneity, methodological limitations, potential non-independence of underlying datasets, limited external validation, and the predominance of pediatric and internally validated studies constrain the generalizability of the reported performance. Findings across data modalities and algorithm classes should therefore be interpreted with caution.

At present, AI is better positioned as an adjunct to established ASD screening and diagnostic pathways than as a standalone diagnostic tool. Further progress will require prospective multicenter validation in representative clinical populations, participant-level evaluation, clearly defined reference diagnoses, prespecified models and operating thresholds, and independent datasets and evaluation procedures. Future studies should also assess calibration, clinical utility, fairness, interpretability, and implementation feasibility to determine whether diagnostic performance can translate into meaningful clinical benefit.

## Data Availability

The original contributions presented in the study are included in the article/Supplementary material, further inquiries can be directed to the corresponding author/s.

## Supplementary material

Supplementary Figure S1 HSROC curve after excluding studies with reviewer-selected best-performing models (*n* = 23). The summary point, 95% confidence region, 95% prediction region, and HSROC AUC with BCa 95% confidence interval are shown.

Supplementary Figure S2 HSROC curve restricted to studies with clearly defined reference standards (*n* = 16). The summary point, 95% confidence region, 95% prediction region, and HSROC AUC with BCa 95% confidence interval are shown.

Supplementary Figure S3 Forest plots of diagnostic performance by age group: **(A)** sensitivity and **(B)** specificity. Pooled estimates are shown for pediatric and adult/mixed-age studies; the unclear-age study is shown descriptively.

Supplementary Figure S4 Forest plots of diagnostic performance by validation strategy: **(A)** sensitivity and **(B)** specificity. Pooled estimates are shown for cross-validation, hold-out validation, and external validation; the other/unclear category is shown descriptively.

Supplementary Figure S5 Forest plots of diagnostic performance by data modality: **(A)** sensitivity and **(B)** specificity. Pooled estimates are shown for clinical/behavioral, eye-tracking/video-audio, EEG, and multimodal studies; the imaging study is shown descriptively.

Supplementary Figure S6 Forest plots of diagnostic performance by algorithm family: **(A)** sensitivity and **(B)** specificity. Pooled estimates are shown for algorithm categories with at least four studies; smaller categories are shown descriptively.

Supplementary Figure S7 HSROC analyses for subgroups with unstable fitted SROC models: **(A)** adult/mixed-age, **(B)** cross-validation, **(C)** hold-out validation, **(D)** eye-tracking/video-audio, **(E)** EEG, **(F)** multimodal, and **(G)** support vector machine. HSROC AUCs were not reported for these subgroups because the fitted SROC models were unstable.

Supplementary Figure S8 Metric-specific odds-ratio contrasts from meta-regression analyses according to **(A)** age group, **(B)** data modality, **(C)** validation strategy, and **(D)** broad algorithm class. Reference groups were pediatric age, clinical/behavioral data, cross-validation, and traditional non-ensemble machine-learning methods, respectively. Points and error bars represent odds ratios with 95% confidence intervals; these metric-specific odds ratios should not be interpreted as diagnostic odds ratios.

Supplementary Figure S9 Leave-one-out sensitivity analysis of overall diagnostic performance: **(A)** pooled sensitivity and **(B)** pooled specificity after sequential omission of each study. The dashed vertical line indicates the corresponding estimate from the complete 26-study dataset.

Supplementary Table S1 Completed PRISMA-DTA checklist.

Supplementary Table S2 Detailed search strategies for PubMed, Embase, Web of Science, the Cochrane Library, and IEEE Xplore.
