# Supplementary material for: Artificial intelligence for detecting autism spectrum disorder: a systematic review and meta-analysis
Source: Front Public Health. 2026 Oct 12;14:1923851. doi: 10.3389/fpubh.2026.1923851 (PMC13710828; doi:10.3389/fpubh.2026.1923851)
Supplement: Supplementary file 2 [file Supplementary_file_2.docx]

**Search strategy (PubMed).**

| **#** | **Query** | **Results** |
| --- | --- | --- |
| 1 | "Autistic Spectrum Disorder"[Mesh] | 49,838 |
| 2 | ((((((((((((((((Disorder, Autistic[Text Word]) OR (Disorders, Autistic[Text Word])) OR (Autism[Text Word])) OR (Autism, Early Infantile[Text Word])) OR (Early Infantile Autism[Text Word])) OR (Infantile Autism, Early[Text Word])) OR (Autism, Infantile[Text Word])) OR (Infantile Autism[Text Word])) OR (Kanner's Syndrome[Text Word])) OR (Kanners Syndrome[Text Word])) OR (Kanner Syndrome[Text Word])) OR (ASD[Text Word])) OR (Autistic Spectrum Disorders[Text Word])) OR (Disorder, Autistic Spectrum[Text Word])) OR (Autism Spectrum Disorders[Text Word])) OR (Pervasive Child Development Disorders[Text Word])) OR (Pervasive Development Disorders[Text Word]) | 88,057 |
| 3 | #1 or #2 | 93,713 |
| 4 | "Algorithms"[Mesh] | 531,599 |
| 5 | (((((((((((((((((((((((((Artificial Intelligence[Text Word]) OR (Deep Learning[Text Word])) OR (Machine Learning[Text Word])) OR (Natural Language Processing[Text Word])) OR (neural network[Text Word])) OR (unsupervised learning[Text Word])) OR (supervised learning[Text Word])) OR (computer aided[Text Word])) OR (convolutional neural network[Text Word])) OR (Recurrent neural network[Text Word])) OR (RNN[Text Word])) OR (CNN[Text Word])) OR (reinforcement learning[Text Word])) OR (feedforward neural network[Text Word])) OR (deep belief network[Text Word])) OR (Boltzmann machine[Text Word])) OR (Autoencoder[Text Word])) OR (multilayer perceptron[Text Word])) OR (convolutional learning[Text Word])) OR (long short-term memory[Text Word])) OR (LSTM[Text Word])) OR (Radiomic[Text Word])) OR (Radiogenomic[Text Word])) OR (random forest[Text Word])) OR (support vector machine[Text Word])) OR (Ensemble[Text Word]) | 495,798 |
| 6 | #4 or #5 | 818,657 |
| 7 | #3 and #6 | 3,220 |

**Search strategy (Web of Science).**

| **#** | **Query** | **Results** |
| --- | --- | --- |
| 1 | TS=(Autistic Spectrum Disorder OR Disorder, Autistic OR Disorders, Autistic OR Autism OR Autism, Early Infantile OR Early Infantile Autism OR Infantile Autism, Early OR Autism, Infantile OR Infantile Autism OR Kanner's Syndrome OR Kanners Syndrome OR ASD OR Autistic Spectrum Disorders OR Disorder, Autistic Spectrum OR Autism Spectrum Disorders OR Pervasive Child Development Disorders OR Pervasive Development Disorders) | 133,983 |
| 2 | TS=(Algorithms OR Artificial Intelligence OR Deep Learning OR Machine Learning OR Natural Language Processing OR neural network OR unsupervised learning OR supervised learning OR computer aided OR convolutional neural network OR Recurrent neural network OR RNN OR CNN OR reinforcement learning OR feedforward neural network OR deep belief network OR Boltzmann machine OR Autoencoder OR multilayer perceptron OR convolutional learning OR long short-term memory OR LSTM OR Radiomic OR Radiogenomic OR random forest OR support vector machine OR Ensemble) | 4,646,445 |
| 3 | #1 AND #2 | 8,330 |

**Search strategy (Cochrane).**

| **#** | **Query** | **Results** |
| --- | --- | --- |
| 1 | MeSH descriptor: [Autistic Spectrum Disorder] explode all trees | 2,960 |
| 2 | (Disorder, Autistic):ti,ab,kw OR (Disorders, Autistic):ti,ab,kw OR (Autism):ti,ab,kw OR (Autism, Early Infantile):ti,ab,kw OR (Early Infantile Autism):ti,ab,kw OR (Infantile Autism, Early):ti,ab,kw OR (Autism, Infantile):ti,ab,kw OR (Infantile Autism):ti,ab,kw OR (Kanner's Syndrome):ti,ab,kw OR (Kanners Syndrome):ti,ab,kw OR (Kanner Syndrome):ti,ab,kw OR (ASD):ti,ab,kw OR (Autistic Spectrum Disorders):ti,ab,kw OR (Disorder, Autistic Spectrum):ti,ab,kw OR (Autism Spectrum Disorders):ti,ab,kw OR (Pervasive Child Development Disorders):ti,ab,kw OR (Pervasive Development Disorders):ti,ab,kw | 7,089 |
| 3 | #1 OR #2 | 7,103 |
| 4 | MeSH descriptor: [Algorithms] explode all trees | 8,591 |
| 5 | (Artificial Intelligence):ti,ab,kw OR (Deep Learning):ti,ab,kw OR (Machine Learning):ti,ab,kw OR (Natural Language Processing):ti,ab,kw OR (neural network):ti,ab,kw OR (unsupervised learning):ti,ab,kw OR (supervised learning):ti,ab,kw OR (computer aided):ti,ab,kw OR (convolutional neural network):ti,ab,kw OR (Recurrent neural network):ti,ab,kw OR (RNN):ti,ab,kw OR (CNN):ti,ab,kw OR (reinforcement learning):ti,ab,kw OR (feedforward neural network):ti,ab,kw OR (deep belief network):ti,ab,kw OR (Boltzmann machine):ti,ab,kw OR (Autoencoder):ti,ab,kw OR (multilayer perceptron):ti,ab,kw OR (convolutional learning):ti,ab,kw OR (long short-term memory):ti,ab,kw OR (LSTM):ti,ab,kw OR (Radiomic):ti,ab,kw OR (Radiogenomic):ti,ab,kw OR (random forest):ti,ab,kw OR (support vector machine):ti,ab,kw OR (Ensemble):ti,ab,kw | 14,942 |
| 6 | #4 OR #5 | 21,044 |
| 7 | #3 AND #6 | 180 |

**Search strategy (Embase).**

| **#** | **Query** | **Results** |
| --- | --- | --- |
| 1 | 'Autistic Spectrum Disorder'/exp | 13,910 |
| 2 | 'disorder, autistic':ab,ti OR 'disorders, autistic':ab,ti OR 'autism':ab,ti OR 'autism, early infantile':ab,ti OR 'early infantile autism':ab,ti OR 'infantile autism, early':ab,ti OR 'autism, infantile':ab,ti OR 'infantile autism':ab,ti OR 'kanners syndrome':ab,ti OR 'kanner syndrome':ab,ti OR 'asd':ab,ti OR 'autistic spectrum disorders':ab,ti OR 'disorder, autistic spectrum':ab,ti OR 'autism spectrum disorders':ab,ti OR 'pervasive child development disorders':ab,ti OR 'pervasive development disorders':ab,ti | 114,407 |
| 3 | #1 OR #2 | 115,874 |
| 4 | 'Algorithms'/exp | 229,099 |
| 5 | 'artificial intelligence':ab,ti OR 'deep learning':ab,ti OR 'machine learning':ab,ti OR 'natural language processing':ab,ti OR 'neural network':ab,ti OR 'unsupervised learning':ab,ti OR 'supervised learning':ab,ti OR 'computer aided':ab,ti OR 'convolutional neural network':ab,ti OR 'recurrent neural network':ab,ti OR 'rnn':ab,ti OR 'cnn':ab,ti OR 'reinforcement learning':ab,ti OR 'feedforward neural network':ab,ti OR 'deep belief network':ab,ti OR 'boltzmann machine':ab,ti OR 'autoencoder':ab,ti OR 'multilayer perceptron':ab,ti OR 'convolutional learning':ab,ti OR 'long short-term memory':ab,ti OR 'lstm':ab,ti OR 'radiomic':ab,ti OR 'radiogenomic':ab,ti OR 'random forest':ab,ti OR 'support vector machine':ab,ti OR 'ensemble':ab,ti | 490,968 |
| 6 | #4 OR #5 | 648,092 |
| 7 | #3 AND #6 | 3,005 |

**Search strategy ( IEEE Xplore).**

| **S** | **Query** | **Results** |
| --- | --- | --- |
| 1 | (("All Metadata":Autistic Spectrum Disorder) OR ("All Metadata":Disorder, Autistic) OR ("All Metadata":Disorders, Autistic) OR ("All Metadata":Autism) OR ("All Metadata":Autism, Early Infantile) OR ("All Metadata":Early Infantile Autism) OR ("All Metadata":Infantile Autism, Early) OR ("All Metadata":Autism, Infantile) OR ("All Metadata":Infantile Autism) OR ("All Metadata":Kanner's Syndrome) OR ("All Metadata":Kanners Syndrome) OR ("All Metadata":Kanner Syndrome) OR ("All Metadata":ASD) OR ("All Metadata":Autistic Spectrum Disorders) OR ("All Metadata":Disorder, Autistic Spectrum) OR ("All Metadata":Autism Spectrum Disorders) OR ("All Metadata":Pervasive Child Development Disorders) OR ("All Metadata":Pervasive Development Disorders)) AND (("All Metadata":Algorithms) OR ("All Metadata":Artificial Intelligence) OR ("All Metadata":Deep Learning) OR ("All Metadata":Machine Learning) OR ("All Metadata":Natural Language Processing) OR ("All Metadata":neural network) OR ("All Metadata":unsupervised learning) OR ("All Metadata":supervised learning) OR ("All Metadata":computer aided) OR ("All Metadata":convolutional neural network) OR ("All Metadata":Recurrent neural network) OR ("All Metadata":RNN) OR ("All Metadata":CNN) OR ("All Metadata":reinforcement learning) OR ("All Metadata":feedforward neural network) OR ("All Metadata":deep belief network) OR ("All Metadata":Boltzmann machine) OR ("All Metadata":Autoencoder) OR ("All Metadata":multilayer perceptron) OR ("All Metadata":convolutional learning) OR ("All Metadata":long short-term memory) OR ("All Metadata":LSTM) OR ("All Metadata":Radiomic) OR ("All Metadata":Radiogenomic) OR ("All Metadata":random forest) OR ("All Metadata":support vector machine) OR ("All Metadata":Ensemble)) | 2,652 |
